# Synthesis of the Benzo-fused Indolizidine Alkaloid Mimics

**DOI:** 10.1186/1860-5397-3-42

**Published:** 2007-11-30

**Authors:** Daniel L Comins, Kazuhiro Higuchi

**Affiliations:** 1 Department of Chemistry, North Carolina State University, Raleigh, North Carolina 27695-8204, USA; 2 Meiji Pharmaceutical University, 2-522-1 Noshio, Kiyose, Tokyo 204-8588, Japan

## Abstract

A general synthesis of various benzo-fused indolizidine alkaloid mimics has been developed. The indolizidine derivatives **8** were prepared via heteroaryl Grignard addition to *N*-acylpyridinium salts followed by an intramolecular Heck cyclization. Further substitution reactions were developed to demonstrate that heterocycles **8** are good scaffolds for chemical library preparation.

## Background

**Figure 1:**
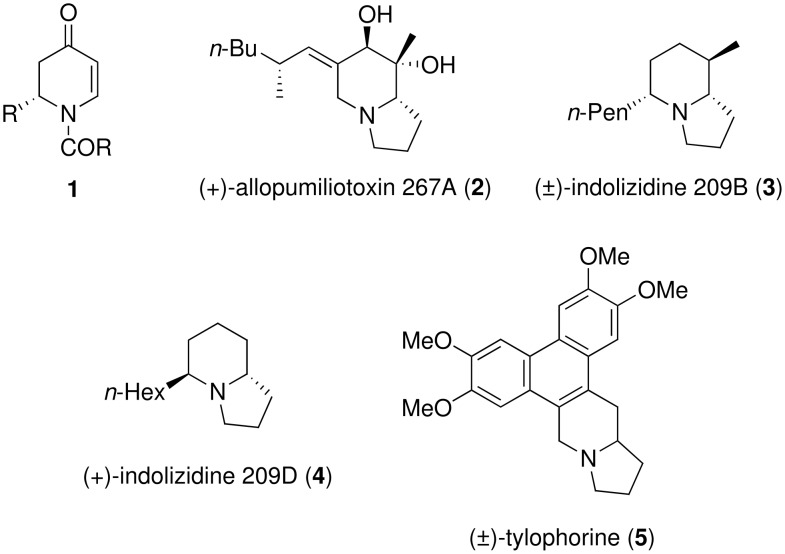
*N*-Acyldihydropyridone **1** and indolizidine alkaloids.

As part of a program directed at studying the synthesis and synthetic utility of *N*-acyldihydropyridones, the heterocycles **1** were developed as useful building blocks for alkaloid synthesis (Figure 1). [1–2] Biologically active indolizidine alkaloids [3] such as (+)-allopumiliotoxin 267A (**2**) [4], (±)-indolizidine 209B (**3**) [5], (+)-indolizidine 209D (**4**) [6], and (±)-tylophorine (**5**) [7] were prepared in racemic or enantiopure form using these dihydropyridone intermediates. Herein we demonstrate the utility of this chemistry for preparing diverse benzo-fused indolizidine compounds.

## Results and Discussion

The reaction of various kinds of heteroaryl Grignard reagents with the *N*-acylpyridinium salt prepared from 4-methoxypyridine (**6**) and 2-iodobenzoylchloride (**7a**) was studied (Table 1). The addition of 2-furyl [8], 2-thienyl [9] and 2-pyrrolyl [10–11] Grignard reagents gave *N*-acyldihydropyridones **1a-c** in good yields (entries 1–3). In addition, the *N*-methyl-2-indolyl [11] Grignard reagent gave **1d** in moderate yield (entry 4). In spite of trying various methods of preparing the 2-pyridyl [12–15] Grignard reagent, **1e** was obtained in only 15% yield (entry 5). Encouraged by these results, the reaction of 3-heteroaryl Grignard reagents was also examined (entries 6–9). The 3-furyl [16] and 3-thienyl [17] Grignard reagents were prepared from the corresponding 3-bromo compounds and gave **1f** and **1g** in moderate yields (entries 6,7). The compounds **1h** and **1i** were prepared in good yield from *N*-TIPS-3-bromopyrrole [18] and *N*-TIPS-3-bromoindole (entries 8,9).

**Table 1:** Reaction with 2- and 3-substituted heteroaryl Grignard reagents

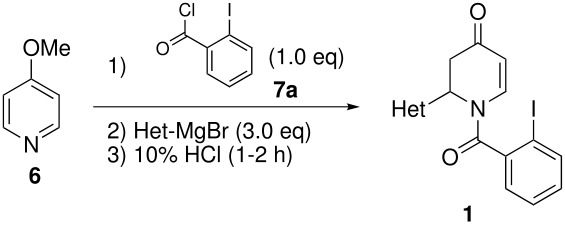					

entry	Het-MgBr	Yield of **1**	entry	Het-MgBr	Yield of **1**

1	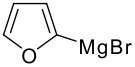	**1a** 83%	6	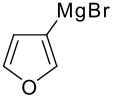	**1f** 55%
2	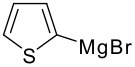	**1b** 86%	7	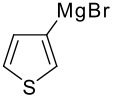	**1g** 58%
3	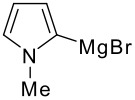	**1c** 69%	8	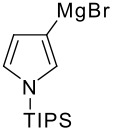	**1h** 9%
4	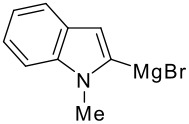	**1d** 51%	9	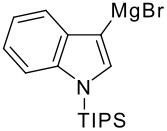	**1i** 77%
5	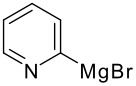	**1e** 15%			

Next, the intramolecular reductive Heck cyclization with *N*-acyl-2,3-dihydropyridones **1a-i** was investigated (Table 2). A short synthesis of indolizidine alkaloids of type **8** by using Heck or anionic cyclization methods was developed. [6,19] In this reaction, only the trans diastereomer was obtained as determined by analysis of the ^1^H-NMR spectrum of the crude product. This methodology is useful for the synthesis of various types of indolizidine alkaloids and their mimics. Treatment of **1a-i** with 5 mol% of palladium catalyst, 2 equiv of formic acid and 4 equiv of triethylamine at 80°C in DMF provided **8a-i** in good yields. THF could also be used as a solvent in this reaction. In the case of **1h** and **1i**, the *N*-TIPS group was cleaved under the reaction conditions (entries 8,9).

**Table 2:** Intramolecular reductive Heck cyclization

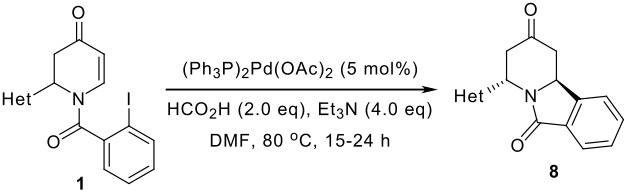					

entry	**1**	yield of **8**	entry	**1**	yield of **8**

1	**1a**	**8a** 82%	6	**1f**	**8f** 78%
2	**1b**	**8b** 81%	7	**1g**	**8g** 79%
3	**1c**	**8c** 74%	8	**1h**	**8h** 57%^a^
4	**1d**	**8d** 48%	9	**1i**	**8i** 82%^a^
5	**1e**	**8e** 31%			

*a* TIPS group was cleaved.

**Scheme 1:**
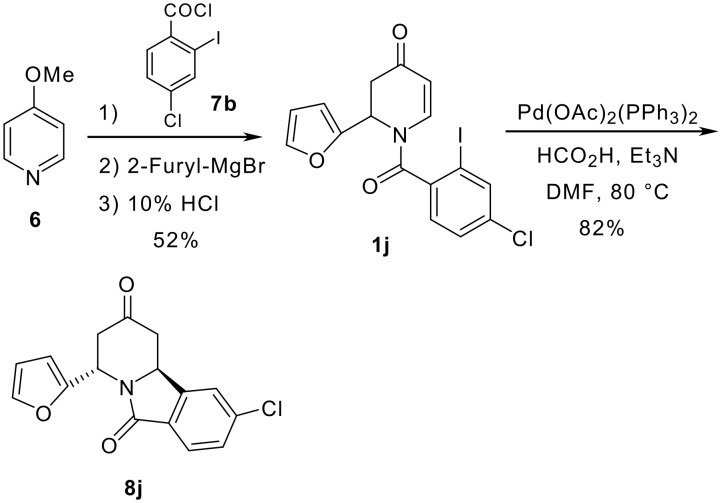
Preparation of chloro-substituted compound **8j**.

To add more points of diversity, the preparation of derivatives containing functionality in the benzene ring was examined. The chloro-substituted compound **1j** was prepared from **6** and 4-chloro-2-iodobenzoylchloride (**7b**). [20] The reductive Heck cyclization of **1j** proceeded without difficulty to provide compound **8j** in 82% yield (Scheme 1).

**Scheme 2:**
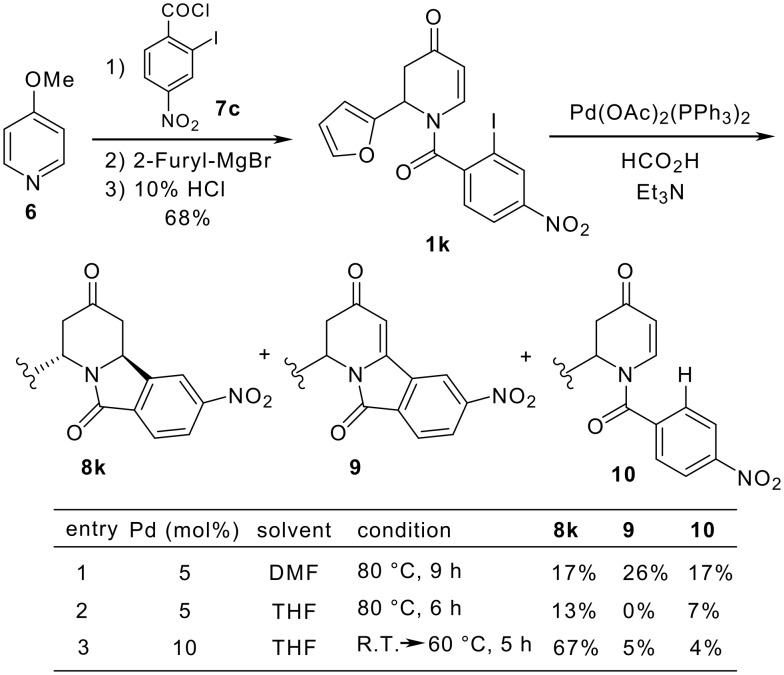
Preparation of nitro-substituted compound **8k**.

Next, the nitro-substituted compound **1k** was prepared from 4-methoxypyridine (**6)** and 2-iodo-4-nitrobenzoyl chloride (**7c**) (Scheme 2). [21] Although the reductive Heck cyclization of **1k** gave the desired compound **8k** in 17% yield, the non-reductive cyclized product **9** and uncyclized compound **10** were isolated in 26% and 17%, respectively (entry 1). The reaction in THF with 10 mol% of palladium catalyst at a lower reaction temperature gave **8k** in 67% yield (entry 3). [22]

**Scheme 3:**
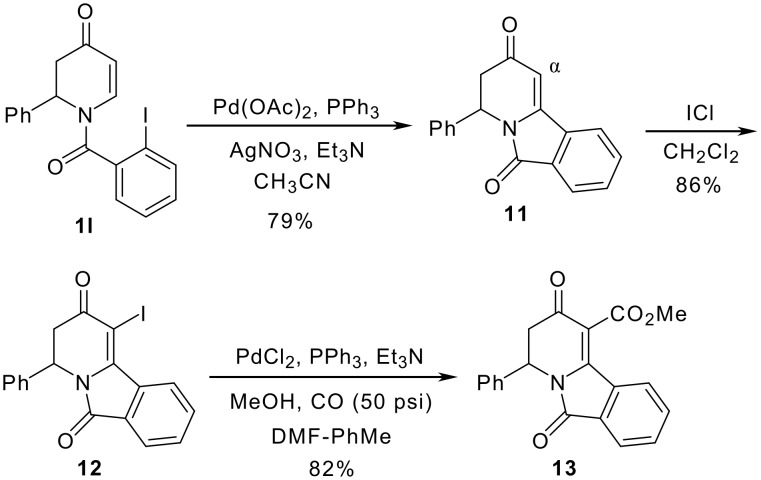
α-Methoxycarbonylation of **11**.

Scheme 3 shows a method for substitution at the α-position of *N*-acyldihydropyridone **11**. Our laboratories have reported C-5 substitution of 5-iodo-1,2-dihydropyridones via palladium mediated cross-coupling and carboalkoxylation. [23] Initially, non-reductive Heck cyclization of **1l** [24] was carried out in the presence of Pd(OAc)_2_ and AgNO_3_ in CH_3_CN. [22] Treatment of the product **11** with ICl in CH_2_Cl_2_ at 0°C gave the iodinated dihydropyridone **12** in 86% yield. Palladium-catalyzed carboalkoxylation reaction of **12** gave the α-methoxycarbonyl dihydropyridone **13** in 82% yield.

**Scheme 4:**
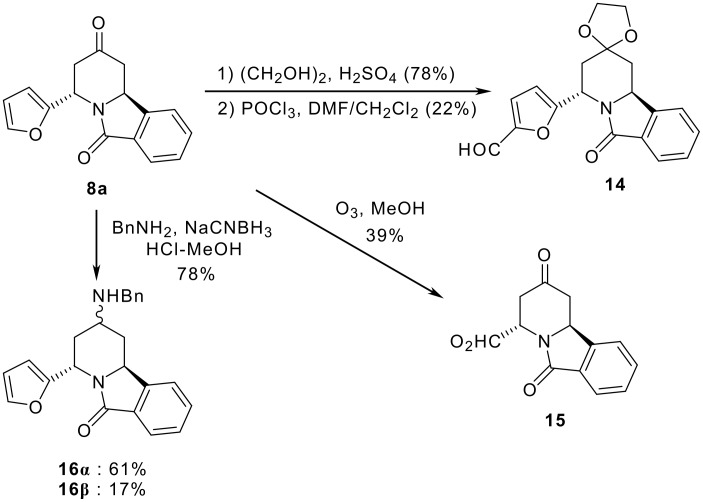
Modification of functional groups within **8a**.

The addition and modification of functional groups on **8a** were investigated (Scheme 4). The protection of the C-4 carbonyl of **8a** as a ketal followed by Vilsmeier-Haack formylation [25] furnished **14** in 22% yield. The furan ring of **8a** was converted to a carboxylic acid by ozonolysis to afford **15**. The reductive amination of **8a** with benzylamine provided **16α** and **16β** in good yield. The stereochemistry of these compounds was determined by NOESY NMR analysis. These functional groups, such as carboxylic acid and secondary amine, provide diversity which could be important for the development of biologically active derivatives.

## Conclusion

The synthesis and chemistry of indolizidine derivatives **8** was investigated with the goal of providing access to diverse heterocyclic compounds of potential biological activity. The various kinds of *N*-acyldihydropyridones **1** were conveniently prepared from heteroaryl Grignard reagents and *N*-acylpyridinium salts. Subsequently, dihydropyridones **1** were converted to **8** by use of an intramolecular Heck cyclization. The chloro- and nitro-substituted acyl chlorides **7** were also used to provide compounds with additional synthetic handles. The α-position of dihydropyridone **11** was halogenated and carbonylated to provide ester **13**. Compound **8a** was also converted to furylaldehyde **14**, carboxylic acid **15** and secondary amines **16**. Indolizidine alkaloids such as type **8** are readily synthesized in 2 steps from commercially available compounds. We have demonstrated that compound **8** can be substituted with functional groups, and provide useful scaffolds for the preparation of indolizidine alkaloid mimics.

## Supporting Information

File 1 Experimental Section. Experimental details and full spectroscopic data for new compounds
