# Supplementary material for: Synthesis of the Benzo-fused Indolizidine Alkaloid Mimics
Source: Beilstein J Org Chem. 2007 Nov 30;3:42. doi: 10.1186/1860-5397-3-42 (PMC2200666; doi:10.1186/1860-5397-3-42)
Supplement: File 1 — Experimental Section. Experimental details and full spectroscopic data for new compounds [file Beilstein_J_Org_Chem-03-42-s001.doc]

**Synthesis of the Benzo-fused Indolizidine Alkaloid Mimics**

Daniel L. Comins*^1^ and Kazuhiro Higuchi^2^

^1^Department of Chemistry, North Carolina State University, Raleigh, North Carolina 27695-8204 USA

^2^Meiji Pharmaceutical University, 2-522-1 Noshio, Kiyose, Tokyo 204-8588 JAPAN

Email: Daniel L. Comins* -daniel_comins@ncsu.edu; Kazuhiro Higuchi -khiguchi@my-pharm.ac.jp

**General Experimental Section:** All reactions described in this section were performed using oven-dried glassware under an argon or dry nitrogen atmosphere. THF, Et_2_O and toluene were dried by distillation using general methods. Other solvents were stored over molecular sieves under argon or nitrogen and used directly. In the work up procedure, the extracts were washed with brine and dried over Na_2_SO_4_ or MgSO_4_. Purification of the crude product was performed with radial PLC or column chromatography on silica gel. ^1^H and ^13^C NMR spectra were acquired in deuterated chloroform (CDCl_3_).

General synthetic procedures for *N*-acyl-2,3-dihydropyridones **1** were described in our former report. [1] Preparations of the heterocyclic Grignard reagents were carried out using literature procedures. See; 2-furyl [2], 2-thienyl [3], *N*-methyl-2-pyrrolyl [4,5], *N*-methyl-2-indolyl [4,5], 2-pyridyl [6-9], 3-furyl [10], 3-thienyl [11], *N*-TIPS-3-pyrrolyl [12], *N*-TIPS-3-indolyl [13].

**2-(2-Furyl)-1-(2-iodobenzoyl)-2,3-dihydro-4-pyridone (1a)**

White amorphous solid (EtOAc/hexanes = 1/2–1/1): mp 101–103 °C; IR (NaCl) 1666, 1600, 1342, 1292, 1217, 1152 cm^–1^; ^1^H NMR shows indefinite rotamers. Anal. Calcd for C_16_H_12_INO_3_: C, 48.88; H, 3.08; N, 3.56. Found: C, 48.91; H, 3.10; N, 3.58.

**1-(2-Iodobenzoyl)-2-(2-thienyl)-2,3-dihydro-4-pyridone (1b)**

White amorphous solid (EtOAc/hexanes = 1/3–1/1): mp 127–129 °C; IR (NaCl) 1666, 1599, 1336, 1293, 1217, 1156 cm^–1^; ^1^H NMR shows indefinite rotamers. Anal. Calcd for C_16_H_12_INO_2_S: C, 46.96; H, 2.96; N, 3.42. Found: C, 46.99; H, 3.00; N, 3.53.

**1-(2-Iodobenzoyl)-2-(1-methyl-2-pyrrolyl)-2,3-dihydro-4-pyridone (1c)**

White-yellow amorphous solid (EtOAc/hexanes = 1/2–1/1): mp 187–189 °C; IR (NaCl) 1667, 1600, 1339, 1300, 1223, 1153 cm^–1^; ^1^H NMR shows indefinite rotamers. HRMS calcd for C_17_H_15_IN_2_O_2_ 407.0257 (M+H)^+^, found 407.0246.

**1-(2-Iodobenzoyl)-2-(1-methyl-2-indolyl)-2,3-dihydro-4-pyridone (1d)**

White solid (EtOAc/hexanes = 1/2–1/1): mp 209–211 °C; IR (NaCl) 1663, 1598, 1466, 1338, 1216, 1153 cm^–1^; ^1^H NMR shows indefinite rotamers. Anal. Calcd for C_21_H_17_IN_2_O_2_: C, 55.28; H, 3.76; N, 6.14. Found: C, 55.21; H, 3.85; N, 6.06.

**1-(2-Iodobenzoyl)-2-(2-pyridyl)-2,3-dihydro-4-pyridone (1e)**

Yellow oil (EtOAc/hexanes = 1/5–1/1): IR (NaCl) 1739, 1671, 1606, 1453, 1328, 1220, 1190, 1179 cm^–1^; ^1^H NMR shows indefinite rotamers. HRMS calcd for C_17_H_13_IN_2_O_2_ 405.0100 (M+H)^+^, found 405.0104.

**2-(3-Furyl)-1-(2-iodobenzoyl)-2,3-dihydro-4-pyridone (1f)**

Brown oil (EtOAc/hexanes = 1/3–1/2): IR (NaCl) 1664, 1596, 1340, 1304, 1234, 1152 cm^–1^; ^1^H NMR shows indefinite rotamer. HRMS calcd for C_16_H_12_IN_2_O_3_ 393.9940 (M+H)^+^, found 393.9936.

**1-(2-Iodobenzoyl)-2-(3-thienyl)-2,3-dihydro-4-pyridone (1g)**

White amorphous solid (EtOAc/hexanes = 1/4–1/2): IR (NaCl) 1664, 1597, 1340, 1294, 1205, 1156 cm^–1^; ^1^H NMR shows indefinite rotamers. Anal. Calcd for C_16_H_12_IN_2_O_2_S: C, 46.96; H, 2.96; N, 3.42. Found: C, 47.12; H, 2.99; N, 3.33.

**1-(2-Iodobenzoyl)-2-(1-TIPS-3-pyrrolyl)-2,3-dihydro-4-pyridone (1h)**

Brown oil (EtOAc/hexanes = 1/5): IR (NaCl) 1668, 1598, 1337, 1153, 1094 cm^–1^; ^1^H NMR shows indefinite rotamers. Anal. Calcd for C_25_H_33_IN_2_O_2_Si: C, 54.74; H, 6.06; N, 5.11. Found: C, 54.73; H, 6.12; N, 5.03.

**1-(2-Iodobenzoyl)-2-(1-TIPS-3-indolyl)-2,3-dihydro-4-pyridone (1i)**

Colorless oil (EtOAc/hexanes = 1/5–1/3): IR (NaCl) 1666, 1598, 1453, 1336, 1221 cm^–1^; ^1^H NMR shows indefinite rotamers. Anal. Calcd for C_29_H_35_IN_2_O_2_Si: C, 58.19; H, 5.89; N, 4.68. Found: C, 58.29; H, 5.92; N, 4.57.

**1-(4-Chloro-2-iodobenzoyl)-2-(2-furyl)-2,3-dihydro-4-pyridone (1j)**

Light brown oil (EtOAc/hexanes = 1/3): IR (NaCl) 1669, 1601, 1338, 1223, 1150cm^–1^; ^1^H NMR shows indefinite rotamers. HRMS calcd for C_16_H_11_ClINO_3_ 427.9560 (M+H)^+^, found 427.9565.

**2-(2-Furyl)-1-(4-nitro-2-iodobenzoyl)-2,3-dihydro-4-pyridone (1k)**

Light brown oil (EtOAc/hexanes = 1/2–2/3): IR (NaCl) 1667, 1603, 1524, 1341, 1225, 1153 cm^–1^; ^1^H NMR shows indefinite rotamers. HRMS calcd for C_16_H_11_IN_2_O_5_ 438.9791 (M+H)^+^, found 438.9777.

**General synthetic procedure for 8:** A mixture of *N*-acyl-2,3-dihydropyridone **1** (0.2 mmol), Pd(OAc)_2_(PPh_3_)_2_ (0.01 mmol), HCO_2_H (0.4 mmol) and Et_3_N (0.8 mmol) in DMF (2.0 mL) was stirred at 80 °C for 15 h. After concentration of the solvent, the crude material was purified by chromatography to give **8**.

***trans*-4-(2-Furyl)-1,3,4,10b-tetrahydropyrido[2,1-*a*]isoindole-2,6-dione (8a)**

Colorless solid (EtOAc/hexanes = 1/2–1/1): mp 157–159 °C; IR (NaCl) 1692, 1402 cm^–1^; ^1^H NMR (400 MHz) δ 2.29 (dd, *J* = 12.0, 14.4 Hz, 1 H), 2.85 (q, *J* = 7.2 Hz, 1 H), 2.93 (dt, *J* = 14.8, 1.6 Hz, 1 H), 3.06 (ddd, *J* = 2.0, 4.4, 14.4 Hz, 1 H), 4.73 (dd, *J* = 4.0, 11.6 Hz, 1 H), 6.12 (dd, *J* = 1.2, 7.6 Hz, 1 H), 6.29–6.32 (m, 2 H), 7.35–7.41 (m, 2 H), 7.50–7.61 (m, 2 H), 7.93 (d, *J* = 7.2 Hz, 1 H); ^13^C NMR (75 MHz) δ 42.4, 45.0, 45.9, 54.9, 108.1, 110.3, 121.8, 124.3, 128.8, 131.3, 132.1, 142.9, 144.5, 151.9, 165.9, 204.4; Anal. Calcd for C_16_H_13_NO_3_: C, 71.90; H, 4.90; N, 5.24. Found: C, 71.74; H, 5.05; N, 5.14.

***trans*-4-(2-Thienyl)-1,3,4,10b-tetrahydropyrido[2,1-*a*]isoindole-2,6-dione (8b)**

White solid (EtOAc/hexanes = 1/3–1/2): mp 178–180 °C; IR (NaCl) 1692, 1594, 1468, 1400, 1332, 1228 cm^–1^; ^1^H NMR (400 MHz) δ 2.31 (dd, *J* = 14.0, 12.8 Hz, 1 H), 2.95 (qd, *J* = 7.2, 0.8 Hz, 1 H), 3.20 (qd, *J* = 2.0, 14.0, 1 H), 3.11 (dt, *J* = 14.4, 2.0 Hz, 1 H), 4.73 (dd, *J* = 4.4, 12.0 Hz, 1 H), 6.31 (d, *J* = 7.2 Hz, 1 H), 6.93 (dd, *J* = 3.2, 4.8 Hz, 1 H), 6.97–6.99 (m, 1 H), 7.25 (dd, *J* = 1.2, 5.2 Hz, 1 H), 7.39 (dd, *J* = 1.2, 7.6 Hz, 1 H), 7.51–7.61 (m, 2H), 7.93 (dt, *J* = 8.0, 0.8 Hz, 1 H); ^13^C NMR (75 MHz) δ 44.1, 46.0, 46.8, 54.4, 121.9, 124.4, 125.7, 126.1, 127.0, 128.9, 131.1, 132.2, 143.0, 144.4, 165.9, 204.8; Anal. Calcd for C_16_H_13_NO_2_S: C, 67.82; H, 4.62; N, 4.94. Found: C, 67.76; H, 4.74; N, 4.85.

***trans*-4-(1-Methyl-2-pyrrolyl)-1,3,4,10b-tetrahydropyrido[2,1-*a*]isoindole-2,6-dione (8c)**

White solid (EtOAc/CH_2_Cl_2_ = 1/10): mp 203–205 °C; IR (NaCl) 1717, 1686, 1468, 1398, 1299, 1228 cm^–1^; ^1^H NMR (300 MHz) δ 2.21 (ddd, *J* = 0.6, 12.3, 14.7 Hz, 1 H), 2.83 (ddd, *J* = 1.2, 7.5, 15.3 Hz, 1 H), 2.99–3.07 (m, 2 H), 3.61 (s, 3 H), 4.56 (dd, *J* = 4.5, 12.0 Hz, 1 H), 6.03 (dd, *J* = 2.7, 3.9 Hz, 1 H), 6.07–6.12 (m, 2 H), 6.58 (dd, *J* = 1.8, 2.7 Hz, 1H), 7.34–7.38 (m, 1 H), 7.48–7.60 (m, 2 H), 7.88–7.92 (m, 1 H); ^13^C NMR (75 MHz) δ 34.2, 43.3, 43.4, 46.3, 53.8, 106.9, 109.0, 121.9, 123.7, 124.3, 128.8, 129.9, 131.3, 132.1, 144.9, 165.6, 205.1; Anal. Calcd for C_17_H_16_N_2_O_2_: C, 72.84; H, 5.75; N, 9.99. Found: C, 72.80; H, 5.61; N, 9.99.

***trans*-4-(1-Methyl-2-indolyl)-1,3,4,10b-tetrahydropyrido[2,1-*a*]isoindole-2,6-dione (8d)**

Yellow oil (EtOAc/hexanes = 1/3): IR (NaCl) 1714, 1685, 1469, 1405, 1225 cm^–1^; ^1^H NMR (300 MHz) δ 2.29 (dd, *J* = 12.9, 14.7 Hz, 1 H), 2.93–3.13 (m, 3 H), 3.74 (s, 3 H), 4.47 (dd, *J* = 4.2, 11.7 Hz, 1 H), 6.40 (d, *J* = 6.9 Hz, 1 H), 7.00–7.10 (m, 2 H), 7.18–7.27 (m, 3 H), 7.45–7.53 (m, 2 H), 7.77 (dd, *J* = 0.9, 8.4 Hz, 1 H), 7.91–7.95 (m, 1 H); ^13^C NMR (75 MHz) δ 32.8, 43.9, 44.0, 46.4, 54.1, 109.1, 113.9, 119.8, 119.9, 121.7, 122.5, 124.2, 126.3, 127.3, 128.7, 131.7, 131.8, 137.1, 144.6, 165.9, 206.8; HRMS calcd for C_21_H_18_N_2_O_2_ 330.1368 (M+H)^+^, found 330.1385.

***trans*-4-(2-Pyridyl)-1,3,4,10b-tetrahydropyrido[2,1-*a*]isoindole-2,6-dione (8e)**

Yellow oil (EtOAc/hexanes = 2/3–1/1): IR (NaCl) 1716, 1682, 1469, 1439, 1402 cm^–1^; ^1^H NMR (400 MHz) δ 2.28 (dd, *J* = 11.6, 14.8 Hz, 1 H), 2.78 (dd, *J* = 7.2, 14.8 Hz, 1 H), 2.99 (dt, *J* = 13.6, 2.0 Hz, 1 H), 3.18 (ddd, *J* = 1.6, 4.8, 15.2 Hz, 1 H), 5.10 (dd, *J* = 4.8, 12.0 Hz, 1 H), 6.09 (dd, *J* = 1.6, 7.2 Hz, 1 H), 7.20 (dd, *J* = 4.8, 7.6 Hz, 1 H), 7.41 (dd, *J* = 4.8, 7.6 Hz, 2 H), 7.50 (t, *J* = 7.2Hz, 1 H), 7.57 (dt, *J* = 1.2, 7.2 Hz, 1 H), 7.67 (dt, *J* = 1.6, 8.0 Hz, 1 H), 7.89 (d, *J* = 7.6 Hz, 1 H), 8.55 (d, *J* = 4.8 Hz, 1 H); ^13^C NMR (75 MHz) δ 42.8, 45.5, 51.8, 54.9, 121.8, 122.5, 122.9, 124.2, 128.6, 131.4, 132.0, 137.3, 145.2, 149.3, 158.2, 166.3, 204.4; HRMS calcd for C_17_H_14_N_2_O_2_ 279.1344 (M+H)^+^, found 279.1132.

***trans*-4-(3-Furyl)-1,3,4,10b-tetrahydropyrido[2,1-*a*]isoindole-2,6-dione (8f)**

Colorless oil (EtOAc/hexanes = 1/2): IR (NaCl) 1717, 1690, 1468, 1404, 1224, 1154 cm^–1^; ^1^H NMR (400 MHz) δ 2.29 (dd, *J* = 12.0, 14.0 Hz, 1 H), 2.84–2.93 (m, 2 H), 2.99 (ddd, *J* = 1.6, 4.4, 14.4 Hz, 1 H), 4.63 (dd, *J* = 4.4, 12.0 Hz, 1 H), 6.03 (dd, *J* = 2.4, 5.6 Hz, 1 H), 6.30 (t, *J* = 1.2 Hz, 1 H), 7.37–7.40 (m, 3 H), 7.51–7.60 (m, 2 H), 7.93 (dd, *J* = 1.2, 7.6 Hz, 1 H); ^13^C NMR (100 MHz) δ 43.5, 43.8, 46.1, 54.4, 109.5, 121.9, 124.3, 124.8, 128.9, 131.4, 132.1, 140.1, 144.1, 144.4, 166.0, 205.3; HRMS calcd for C_16_H_13_NO_3_ 268.0974 (M+H)^+^, found 268.0959.

***trans*-4-(3-Thienyl)-1,3,4,10b-tetrahydropyrido[2,1-*a*]isoindole-2,6-dione (8g)**

Colorless oil (EtOAc/hexanes = 1/3): IR (NaCl) 1692, 1468, 1402, 1430 cm^–1^; ^1^H NMR (300 MHz) δ 2.31 (ddd, *J* = 0.6, 12.0, 14.1 Hz, 1 H), 2.91 (ddd, *J* = 0.9, 7.5, 14.4 Hz, 1 H), 3.00 (ddd, *J* = 1.5, 4.5, 14.1 Hz, 1 H), 3.09 (dt, *J* = 14.7, 1.8 Hz, 1 H), 4.56 (dd, *J* = 4.2, 12.0 Hz, 1 H), 6.15 (d, *J* = 7.5 Hz, 1 H), 7.01 (dd, *J* = 1.5, 4.8 Hz, 1 H), 7.18–7.20 (m, 1 H), 7.29 (q, *J* = 3.0 Hz, 1 H), 7.35–7.38 (m, 1 H), 7.49–7.61 (m, 2 H), 7.90–7.95 (m, 1 H); ^13^C NMR (75 MHz) δ 43.5, 46.1, 46.8, 54.5, 121.9, 122.6, 124.3, 126.9, 127.1, 128.9, 131.4, 132.1, 140.7, 144.4, 166.0, 205.6; HRMS calcd for C_16_H_13_NO_2_S 284.0754 (M+H)^+^, found 284.0735.

***trans*-4-(3-Pyrrolyl)-1,3,4,10b-tetrahydropyrido[2,1-*a*]isoindole-2,6-dione (8h)**

White solid (EtOAc/hexanes = 1/2–1/1): mp 232–234 °C; IR (NaCl) 3312, 1717, 1674, 1467, 1414, 1222 cm^–1^; ^1^H NMR (400 MHz) δ 2.27 (dd, *J* = 12.0, 14.0 Hz, 1 H), 2.85 (dd, *J* = 7.2, 14.8 Hz, 1 H), 2.94–3.01 (m, 2 H), 4.67 (dd, *J* = 4.0, 11.6 Hz, 1 H), 6.09–6.13 (m, 2 H), 6.73 (t, *J* = 2.4 Hz, 2 H), 7.35 (d, *J* = 6.8 Hz, 1 H), 7.49–7.58 (m, 2 H), 7.92 (d, *J* = 6.4 Hz, 1 H), 8.23 (brs, 1 H); ^13^C NMR (75 MHz) δ 44.1, 45.2, 46.2, 54.3, 107.6, 116.2, 119.0, 121.8, 123.0, 124.2, 128.7, 131.8, 131.9, 144.6, 165.9, 206.4; Anal. Calcd for C_16_H_14_N_2_O_2_: C, 72.16; H, 5.30; N, 10.52. Found: C, 72.02; H, 5.40; N, 10.50.

***trans*-4-(3-Indolyl)-1,3,4,10b-tetrahydropyrido[2,1-*a*]isoindole-2,6-dione (8i)**

White solid (EtOAc/hexanes = 1/2–1/1): mp 253–255 °C; IR (NaCl) 3398, 1674, 1409, 1221 cm^–1^; ^1^H NMR (400 MHz) δ 2.31 (ddd, *J* = 0.8, 16.0, 18.8 Hz, 1 H), 2.94–3.02 (m, 2 H), 3.14 (dt, *J* = 19.2, 2.0 Hz, 1 H), 4.46 (dd, *J* = 15.2, 5.2 Hz, 1 H), 6.42 (d, *J* = 9.6 Hz, 1 H), 7.04–7.10 (m, 1 H), 7.15–7.28 (m, 3 H), 7.34 (dt, *J* = 7.2, 1.2 Hz, 1 H), 7.46–7.54 (m, 2 H), 7.79 (m, 1 H), 7.92–7.96 (m, 1 H), 8.27 (bs, 1 H); ^13^C NMR (75 MHz) δ 43.9, 44.0, 46.4, 54.2, 111.1, 115.6, 119.7, 120.4, 121.8, 122.9, 123.0, 124.3, 125.8, 128.7, 131.7, 131.9, 136.4, 144.6, 166.0, 206.8; Anal. Calcd for C_20_H_16_N_2_O_2_: C, 75.93; H, 5.10; N, 8.86. Found: C, 75.66; H, 5.05 N, 8.85.

***trans*-9-Chloro-4-(3-furyl)-1,3,4,10b-tetrahydropyrido[2,1-*a*]isoindole-2,6-dione (8j)**

Light brown solid (EtOAc/hexanes = 1/3): mp 187–189 °C; IR (NaCl) 1695, 1612, 1404, 1344, 1280, 1226 cm^–1^; ^1^H NMR (400 MHz) δ 2.30 (dd, *J* = 12.4, 14.4 Hz, 1 H), 2.85 (dd, *J* =7.2, 15.2 Hz, 1 H), 2.93 (dt, *J* = 14.8, 1.6 Hz, 1 H), 3.04 (ddd, *J* = 1.6, 6.0, 14.4 Hz, 1 H), 4.72 (dd, *J* = 4.4, 12.0 Hz, 1 H), 6.08 (d, *J* = 7.6 Hz, 1 H), 6.29–6.32 (m, 2 H), 7.35–7.39 (m, 2 H), 7.50 (dd, *J* = 2.0, 8.4 Hz, 1 H), 7.85 (d, *J* = 8.0 Hz, 1 H); ^13^C NMR (100 MHz) δ 42.4, 45.1, 45.6, 54.6, 108.3, 110.4, 122.5, 125.6, 129.5, 129.8, 138.5, 143.1, 146.0, 151.6, 164.9, 203.7; Anal. Calcd for C_16_H_12_ClNO_3_: C, 63.69; H, 4.01; N, 4.64. Found: C, 63.69; H, 4.10 N, 4.55.

***trans*-9-Nitro-4-(3-furyl)-1,3,4,10b-tetrahydropyrido[2,1-*a*]isoindole-2,6-dione (8k)**

Light brown solid (EtOAc/hexanes = 1/2): mp 192–195 °C; IR (NaCl) 1699, 1531, 1406, 1346, 1279, 1228 cm^–1^; ^1^H NMR (400 MHz) δ 2.34 (dd, *J* = 12.0, 14.4 Hz, 1 H), 2.88 (dd, *J* = 7.2, 15.2 Hz, 1 H), 2.97 (dt, *J* = 15.2, 1.2 Hz, 1 H), 3.17 (ddd, *J* = 1.6, 4.4, 14.4 Hz, 1 H), 4.86 (dd, *J* = 4.4, 12.4 Hz, 1 H), 6.12 (dd, *J* = 2.0, 7.6 Hz, 1 H), 6.33–6.34 (m, 2 H), 7.37 (s, 1 H), 8.09 (d, *J* = 8.4 Hz, 1 H), 8.27 (d, *J* = 1.6 Hz, 1 H), 8.42 (dd, *J* = 1.6, 8.4 Hz, 1 H); ^13^C NMR (75 MHz) δ 42.3, 45.3, 45.4, 55.0, 108.7, 110.5, 117.7, 124.6, 125.6, 136.8, 143.3, 145.3, 150.5, 151.2, 163.7, 202.7. HRMS calcd for C_16_H_12_N_2_O_5_ 313.0824 (M+H)^+^, found 313.0835.

**4-Phenyl-3,4-dihydropyrido[2,1-*a*]isoindole-2,6-dione (11)**

A mixture of **1l** [14] (238.0 mg, 0.59 mmol), Pd(OAc)_2_ (13.3 mg, 0.059 mmol), PPh_3_ (31.0 mg, 0.12 mmol), AgNO_3_ (100.3 mg, 0.59 mmol) and Et_3_N (0.17 mL, 1.18 mmol) in CH_3_CN (6.0 mL) was stirred at 80 °C for 4.5 h. After concentration of the solvent, the crude material was purified by radial PLC (CH_2_Cl_2_/EtOAc = 100/0–10/1) to give **11** (129.0 mg, 79%).

Light yellow solid: mp 184–188 °C; IR (CHCl_3_) 1722, 1667, 1631, 1418, 1223, 1114 cm^–1^; ^1^H NMR (300 MHz) δ 3.00 (d, *J* = 16.0 Hz, 1 H), 3.26 (dd, *J* = 7.9, 16.5 Hz, 1 H), 5.86 (d, *J* = 7.3 Hz, 1 H), 6.10 (s, 1H), 7.20-7.29 (m, 5 H), 7.67-7.80 (m, 3 H), 7.91 (d, *J* = 6.5 Hz, 1 H); ^13^C NMR (75 MHz) δ 42.4, 51.8, 101.9, 122.2, 124.3, 126.1, 128.2, 129.0, 129.8, 132.4, 133.0, 134.2, 139.1, 150.2, 165.6, 192.7; HRMS calcd for C_18_H_13_NO_2_ 275.0946 M^+^, found 275.0945.

**1-Iodo-4-phenyl-3,4-dihydropyrido[2,1-*a*]isoindole-2,6-dione (12)**

A solution of **11** (70.8 mg, 0.26 mmol) and ICl (0.31 mL, 1.0 M in CH_2_Cl_2_, 0.31 mmol) in CH_2_Cl_2_ (2.6 mL) was stirred at 0 °C for 1 h. Saturated aqueous NaHCO_3_ (5.0 mL) was added and the mixture was extracted with CH_2_Cl_2_ (5 mL x 3). The crude material was purified by radial PLC (hexanes/EtOAc = 3/1) to give **12** (81.9 mg, 79%).

Light yellow solid: mp 176–178 °C; IR (NaCl) 1719, 1671, 1574, 1382, 1358, 1334, 1123 cm^–1^; ^1^H NMR (400 MHz) δ 3.35–3.38 (m, 2 H), 5.94 (dd, *J* = 2.4, 6.0 Hz, 1 H), 7.15–7.31 (m, 5 H), 7.70–7.82 (m, 2 H), 7.94 (dd, *J* = 0.4, 3.2 Hz, 1 H), 9.05 (dd, *J* = 0.8, 8.0 Hz, 1 H); ^13^C NMR (75 MHz) δ 40.8, 51.1, 77.9, 124.5, 125.8, 125.9, 128.3, 129.1, 130.0, 132.6, 132.7, 134.6, 138.2, 149.8, 164.6, 187.5; HRMS calcd for C_18_H_12_INO_2_ 401.9991 (M+H)^+^, found 401.9997.

**1-Methoxycarbonyl-4-phenyl-3,4-dihydropyrido[2,1-*a*]isoindole-2,6-dione (13)**

A mixture of **12** (37.3 mg, 0.093 mmol), MeOH (40 μL, 0.93 mmol), PdCl_2_ (0.3 mg, 1.4 μmol), PPh_3_ (0.7 mg, 0.003 mmol) and Et_3_N (16 μL, 0.11 mmol) in toluene-DMF (2:1, 3.0 mL) was stirred under 50 psi of CO at 60 °C for 4 h. After concentration of the solvent, the crude material was purified by radial PLC (hexanes/EtOAc = 2/1) to give **13** (25.5 mg, 82%).

Brown solid: mp 225–227 °C; IR (NaCl) 1724, 1670, 1618, 1405, 1365, 1255, 1124 cm^–1^; ^1^H NMR (400 MHz) δ 3.06 (d, *J* = 17.2 Hz, 1 H), 3.29 (dd, *J* = 7.6, 16.0 Hz, 1 H), 3.98 (s, 3 H), 5.87 (d, *J* = 7.2 Hz, 1 H), 7.21–7.33 (m, 5 H), 7.67–7.71 (m, 2 H), 7.81–7.85 (m, 1 H), 7.90–7.94 (m, 1 H); ^13^C NMR (75 MHz) δ 42.1, 51.6, 53.3, 110.8, 124.7, 125.6, 126.2, 128.6, 129.4, 129.7, 133.0, 133.2, 133.7, 138.5, 149.1, 165.4, 165.6, 189.6. HRMS calcd for C_20_H_15_INO_2_ 334.1079 (M+H)^+^, found 334.1076.

**5-(*trans*-2,6-Dioxo-2-ethylene acetal-1,2,3,4,6,10b-hexahydropyrido[2,1-*a*]isoindol-4-yl)-furan-2-carbaldehyde (14)**

A solution of **8a** (200 mg, 0.75 mmol), ethylene glycol (0.84 mL, 15.0 mmol) and concd H_2_SO_4_ (0.2 mL) in benzene (5 mL) was refluxed for 16 h. The mixture was poured into saturated aqueous NaHCO_3_ (20 mL) and extracted with EtOAc (10 mL x 3). The crude material was purified by radial PLC (hexanes/EtOAc = 1/1) to give the ketal (181.6 mg, 78%).

Colorless oil: IR (NaCl) 1692, 1410, 1140, 1119, 1063 cm^–1^; ^1^H NMR (300 MHz) δ 1.49 (t, *J* = 12.6 Hz, 1 H), 1.98 (dd, *J* = 7.2, 13.8 Hz, 1 H), 2.32 (ddd, *J* = 2.4, 3.6, 12.6 Hz, 1 H), 2.57 (ddd, *J* = 1.8, 2.4, 14.1 Hz, 1 H), 3.93–4.07 (m, 4 H), 4.82 (dd, *J* = 3.6, 12.3 Hz, 1 H), 5.86 (d, *J* = 7.2 Hz, 1 H), 6.07–6.10 (m, 1 H), 6.31 (dd, *J* = 1.8, 3.3 Hz, 1 H), 7.34–7.36 (m, 1 H), 7.40–7.43 (m, 1 H), 7.46–7.60(m, 2 H), 7.90–7.93 (m, 1 H); ^13^C NMR (75 MHz) δ 34.6, 40.5, 45.0, 54.6, 64.3, 64.9, 105.6, 107.0, 110.4, 121.8, 124.1, 128.4, 131.6, 131.7, 141.5, 145.3, 153.4, 166.5; HRMS calcd for C_18_H_17_NO_4_ 312.1236 (M+H)^+^, found 312.1250.

To a solution of DMF (60 μL, 0.76 mmol) in CH_2_Cl_2_ (1.0 mL) cooled to 0 °C was added POCl_3_ (35 μL, 0.38 mmol), and the mixture was stirred at RT for 25 min. To this mixture was added a solution of the ketal (53.6 mg, 0.17 mmol) in CH_2_Cl_2_ (2.0 mL) at 0 °C. After stirring at RT for 20 h, the reaction was quenched by aqueous NaOAc (100 mg/10 mL) and the mixture was refluxed for 20 min. The mixture was extracted with CH_2_Cl_2_ (5 mL x 3). The crude material was purified by radial PLC (EtOAc/hexanes = 1/1) to give **14** (13.1 mg, 22%).

Colorless oil: IR (NaCl) 1693, 1678, 1515, 1469, 1410, 1273, 1240, 1121 cm^–1^; ^1^H NMR (300 MHz) δ  1.54 (t, *J* = 12.6 Hz, 1 H), 1.97 (dd, *J* = 6.9, 13.8 Hz, 1 H), 2.32 (ddd, *J* = 2.1, 3.9, 12.6 Hz, 1 H), 2.73 (dt, *J* = 13.8, 1.5, Hz, 1 H), 3.89–4.10 (m, 4 H), 4.86 (dd, *J* = 3.9, 12.3 Hz, 1 H), 5.92 (d, *J* = 6.9 Hz, 1 H), 6.23 (dd, *J* = 1.2, 3.6 Hz, 1 H), 7.20 (d, *J* = 3.6 Hz, 1 H), 7.45 (d, *J* = 7.2 Hz, 1 H), 7.50–7.56 (m, 1 H), 7.61 (dt, *J* = 1.2, 7.5 Hz, 1 H), 7.93 (d, *J* = 7.5 Hz, 1 H), 9.58 (s, 1 H); ^13^C NMR (75 MHz) δ 34.4, 40.4, 45.6, 54.9, 64.5, 64.7, 106.6, 108.6, 121.9, 1213.3, 124.3, 128.6, 131.1, 132.1, 145.3, 152.1, 161.0, 166.7, 177.1; HRMS calcd for C_19_H_17_NO_5_ 340.1185 (M+H)^+^, found 340.1199.

***trans*-2,6-Dioxo-1,2,3,4,6,10b-hexahydropyrido[2,1-*a*]isoindole-4-carboxylic acid (15)**

A solution of **8a** (28.6 mg, 0.11 mmol) in MeOH (3.0 mL) was stirred while adding a stream of O_3_ at –78 °C for 30 min. Argon gas was bubbled through the mixture at –78 °C for 5 min to remove excess O_3_. The solvent was removed and the residue was dissolved in 10% NaOH (5.0 mL) and washed with Et_2_O (5 mL x 3). The aqueous layer was acidified with 10% HCl and extracted with CH_2_Cl_2_ (5 mL x 3). The crude material was purified by radial PLC (CH_2_Cl_2_/MeOH/AcOH = 80/10/1) to give **15** (10.1 mg, 39%).

White solid: mp 101–104 °C; IR (KBr) 3443, 1717, 1647, 1472, 1423 cm^–1^; ^1^H NMR (400 MHz) δ  2.25 (dd, *J* = 12.4, 14.8 Hz, 1 H), 2.81 (dd, *J* = 8.4, 15.6 Hz, 1 H), 3.01 (dd, *J* = 3.2, 16.4 Hz, 1 H), 3.08 (dd, *J* = 3.6, 15.2 Hz, 1 H), 5.14 (dd, *J* = 4.4, 12.0 Hz, 1 H), 5.51 (dd, *J* = 3.2 , 8.0 Hz, 1 H), 7.44 (d, *J* = 7.6 H, 1 H), 7.55 (t, *J* = 7.6 H, 1 H), 7.64 (t, *J* = 7.6 H, 1 H), 7.93 (d, *J* = 7.6 H, 1 H); HRMS calcd for C_13_H_11_NO_4_ 246.0766 (M+H)^+^, found 246.0779.

***trans*-Benzylamino-4-(2-furyl)-1,3,4,10b-tetrahydro-2*H*-pyrido[2,1-*a*]isoindol-6-one (16)**

A mixture of **8a** (80 mg, 0.30 mmol), benzylamine (0.20 mL, 1.8 mmol), concd HCl/MeOH (1:1, 0.12 mL) and NaCNBH_3_ (11 mg, 0.18 mmol) in absolute MeOH (1.0 mL) was stirred at RT for 53 h. This mixture was cooled to 0 °C and concd HCl (4 drops) and H_2_O (40 mL) were added. The solution was brought to pH >10 with solid NaOH. The mixture was extracted with CH_2_Cl_2_ (5 mL x 3). The crude material was purified by radial PLC (hexanes/EtOAc = 2/1–1/1) to give **16α** (65.4 mg, 61%) and **16β** (18.1 mg, 17%).

**16α**: Yellow solid: mp 96–98 °C; IR (NaCl) 3311, 2921, 1687, 1468, 1409 cm^–1^; ^1^H NMR (400 MHz) δ  1.34 (dt, *J* = 3.2, 12.4 Hz, 1 H), 1.39 (bs, 1 H), 1.93 (ddd, *J* = 4.0, 7.2, 14.8 Hz, 1 H), 2.34–2.40 (m, 1 H), 2.63–2.69 (m, 1 H), 3.24–3.28 (m, 1 H), 3.77 (dd, *J* = 13.6, 35.6 Hz, 2 H), 4.97 (dd, *J* = 3.6, 12.0 Hz, 1 H), 5.73 (d, *J* = 7.2 Hz, 1 H), 6.01–6.04 (m, 1 H), 6.33 (q, *J* = 1.6 Hz, 1 H), 7.15–7.31 (m, 5 H), 7.32–7.35 (m, 2 H), 7.47 (dt, *J* = 0.8, 7.2 Hz, 1 H), 7.54 (dt, *J* = 1.2, 7.6 Hz, 1 H), 7.92 (d, *J* = 7.2 Hz, 1 H); ^13^C NMR (100 MHz) δ 30.9, 36.3, 44.5, 50.5, 51.3, 51.8, 104.7, 110.8, 121.8, 124.0, 126.9, 127.8, 128.0, 128.3, 131.4, 131.8, 140.5, 141.4, 146.6, 154.8, 166.6; HRMS calcd for C_23_H_22_N_2_O_2_ 359.1760 (M+H)^+^, found 359.1768.

**16β**: Clear oil: IR (NaCl) 3310, 2926, 1685, 1468, 1410 cm^–1^; ^1^H NMR (400 MHz) δ  1.05 (dd, *J* = 12.0, 23.6 Hz, 1 H), 1.54–1.63 (m, 2 H), 2.57 (ddd, *J* = 12.0, 5.2, 3.6 Hz, 1 H), 2.62 (dt, *J* = 13.2, 2.0 Hz, 1 H), 3.23 (tt, *J* = 3.6, 11.2 Hz, 1 H), 3.90 (s, 2 H), 4.55 (dd, *J* = 3.2, 12.0 Hz, 1 H), 5.83 (d, *J* = 5.6 Hz, 1 H), 6.08 (d, *J* = 3.2 Hz, 1 H), 6.29 (dd, *J* = 1.6, 3.2 Hz, 1 H), 7.24 –7.58 (m, 9 H), 7.89 (d, *J* = 7.2 Hz, 1 H); ^13^C NMR (75 MHz) δ 34.8, 38.5, 45.3, 50.9, 55.6, 106.7, 110.3, 121.7, 124.1, 127.1, 128.1, 128.3, 128.5, 131.5, 131.8, 140.1, 142.0, 145.4, 153.4, 166.3; HRMS calcd for C_23_H_22_N_2_O_2_ 359.1760 (M+H)^+^, found 359.1785.

# References for Experimental Section

1. Comins DL, Joseph SP, Goehring RR: *J Am Chem Soc* 1994, **116:**4719-4728.
2. Focken T, Charette AB: *Org Lett* 2006, **8:**2985-2988.
3. Kuroda M, Nakayama J, Hoshino M: *Tetrahedron* 1993, **49:**3735-3748.
4. Minato A, Suzuki K, Tamao K, Kumada M: *Tetrahedron Lett* 1984, **25:**83-86.
5. Kuethe JT, Comins DL: *J Org Chem* 2004, **69:**2863-2866.
6. Iida T, Wada T, Tomimoto K, Mase T: *Tetrahedron Lett* 2001, **42:**4841-4844.
7. Inoue A, Kitagawa K, Shinokubo H, Oshima K: *J Org Chem* 2001, **66:**4333-4339.
8. Furukawa N, Shibutani T, Fujihara H: *Tetrahedron Lett* 1987, **28:**5845-5848.
9. Ozawa K, Ishii S, Hatanaka M: *Chem Lett* 1985, 1803-1804.
10. Haarmann H, Eberbach W: *Tetrahedron Lett* 1991, **32:**903-906.
11. Rieke RD, Kim SH, Wu X: *J Org Chem* 1997, **62:**6921-6927.
12. Bumagin NA, Nikitina AF, Beletskaya IP: *Russ J Org Chem* 1994, **30:**1619-1629.
13. Vaillancourt V, Albizati KF: *J Am Chem Soc* 1993, **115:**3499-3502.
14. Beckwith ALJ, Joseph SP, Mayadunne RTA: *J Org Chem* 1993, **58:**4198-4199.
